# Supplementary material for: Glutamine deprivation alters the origin and function of cancer cell exosomes
Source: EMBO J. 2020 Jul 28;39(16):e103009. doi: 10.15252/embj.2019103009 (PMC7429491; doi:10.15252/embj.2019103009)
Supplement: Supplementary file 1 — Appendix [file EMBJ-39-e103009-s001.pdf]

# Appendix

## Glutamine deprivation regulates the origin and function of cancer cell exosomes

Shih-Jung Fan, Benjamin Kroeger, Pauline P. Marie, Esther M. Bridges, John D. Mason,
Kristie McCormick, Christos E. Zois, Helen Sheldon, Nasullah Khalid Alham, Errin Johnson,
Matthew Ellis, M. Irina Stefana, Cláudia C. Mendes, S. Mark Wainwright, Christopher Cunningham,
Freddie C. Hamdy, John F. Morris, Adrian L. Harris, Clive Wilson, Deborah C. I. Goberdhan*

*To whom correspondence should be addressed, e-mail: deborah.goberdhan@dpag.ox.ac.uk

## Table of Contents

**Appendix Figure S1 -** page 2

**Appendix Figure S2 -** page 3

**Appendix Figure S3 -** page 5

**Appendix Figure S4 -** page 6

**Appendix Figure S5 -** page 7

**Appendix Figure S6 -** page 8

**Appendix Figure S7 -** page 9

**Appendix Figure S8 -** page 11

**Appendix Figure S9 -** page 12

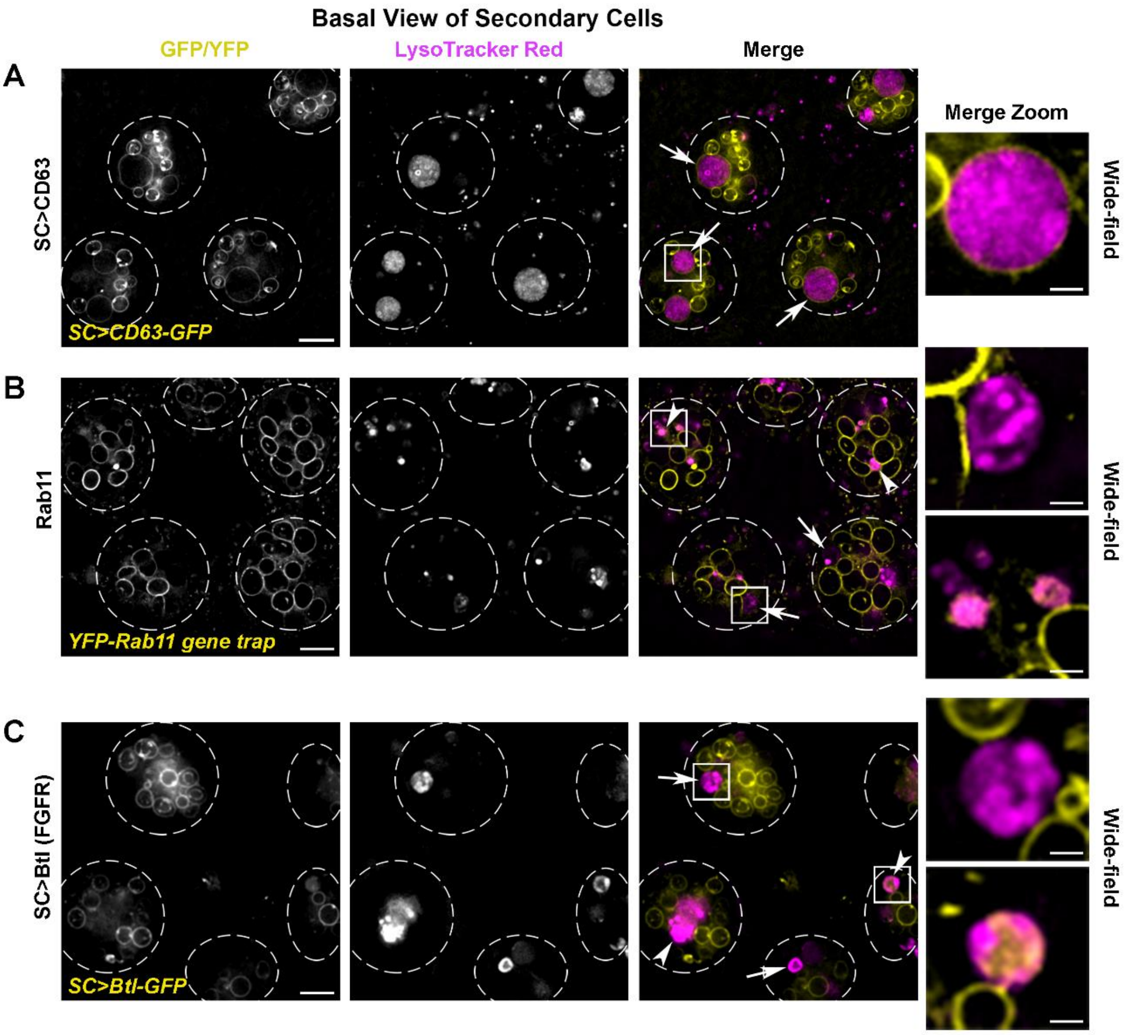


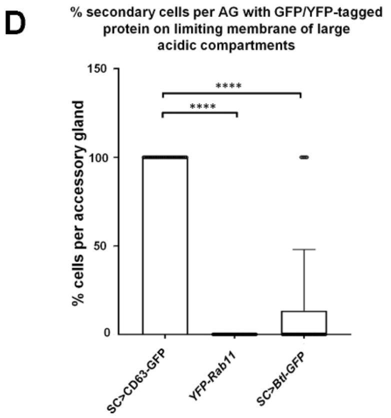


**Appendix Figure S1. YFP-Rab11 and Btl-GFP do not traffic to the limiting membrane of acidic compartments in secondary cells**

Panels **A**-**C** show basal wide-field fluorescence views through living secondary cells (SCs) in same plane as schematic in right panel of Fig 1A. Cells express GFP- and YFP-tagged genes (yellow), with acidic compartments marked by LysoTracker Red® (magenta).

(**A**) CD63-GFP is apparent on the limiting membrane of all large acidic LEL compartments (arrows), when overexpressed in SCs. Boxed region in Merge is magnified in Merge Zoom (see also quantification in **D**).

(**B**) YFP-Rab11 is never found on the limiting membrane of acidic compartments in gene trap males (see also **D**). Boxed regions are magnified in Zoom. Some compartments lack internal YFP-Rab11 (arrows; upper Zoom panel), while others have fluorescent luminal content (arrowheads; lower Zoom panel).

(**C**) Btl-GFP is mostly absent from the limiting membrane of acidic compartments, when overexpressed in SCs (arrows). Boxed regions are magnified in Zoom. Some compartments lack internal Btl-GFP (arrows; upper Zoom panel), while others have fluorescent luminal content (arrowheads; lower Zoom panel).

(**D**) Bar chart showing proportion of SCs containing a large acidic LEL compartment with CD63-GFP, YFP-Rab11 and Btl-GFP on its limiting membrane.

All images are from six-day-old male flies shifted to 29ºC at eclosion. Genotypes of flies carrying multiple transgenes are: *w; P[w$^+$, UAS-CD63-GFP] P[w$^+$, tub-GAL80$^{ts}$]/+; dsx-GAL4/+* (A): *w; P[w$^+$, tub-GAL80$^{ts}$]/+; dsx-GAL4/P[w$^+$, UAS-btl-GFP]* (**C**).

Scale bar in **A**-**C** (10 µm) and in Zoom (2 µm). Values in **D** compared using the Kruskal-Wallis test (n = 30 SCs, 3 per AG). ****P < 0.0001.

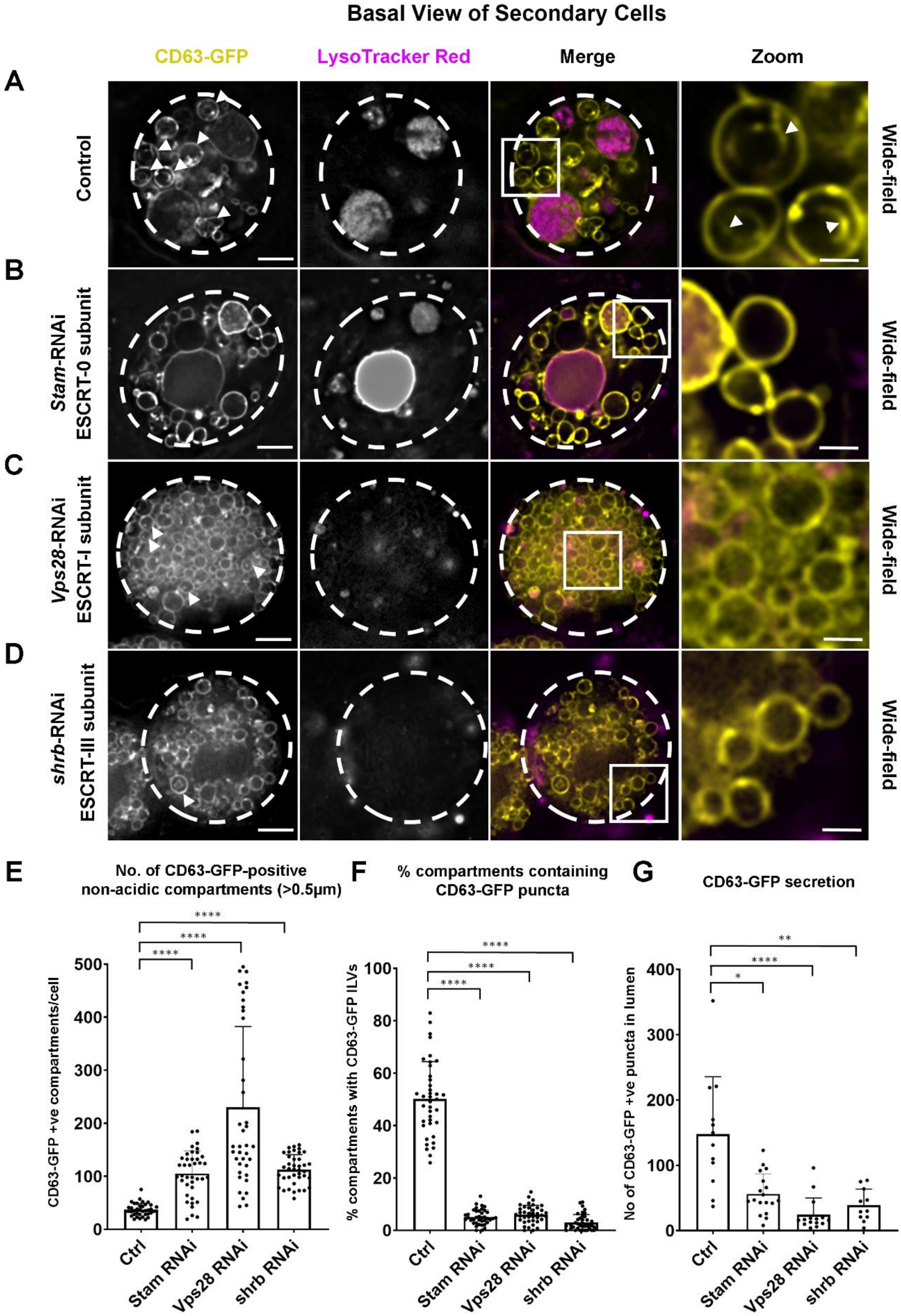

Basal View of Secondary Cells
CD63-GFP
LysoTracker Red
Merge
Zoom
A
Control
Wide-field
B
Stam-RNAi
ESCRT-0 subunit
Wide-field
C
Vps28-RNAi
ESCRT-I subunit
Wide-field
D
shrb-RNAi
ESCRT-III subunit
Wide-field
E
No. of CD63-GFP-positive
non-acidic compartments (>0.5µm)
CD63-GFP +ve compartments/cell
Ctrl
Stam RNAi
Vps28 RNAi
shrb RNAi
F
% compartments containing
CD63-GFP puncta
% compartments with CD63-GFP ILVs
G
CD63-GFP secretion
No of CD63-GFP +ve puncta in lumen

**Appendix Figure S2. ESCRTs regulate exosome biogenesis in the Rab11-positive compartments of *Drosophila* secondary cells**

Panels **A**-**D** show basal wide-field fluorescence views through living secondary cells (SCs) expressing CD63-GFP under the control of SC-specific *dsx*-GAL4. Acidic compartments are marked with LysoTracker Red® (magenta). Boxed non-acidic compartment in Merge is magnified in Zoom.

(**A**) SC with no RNAi expressed (control), as also shown in Fig. 1B. Arrowheads in left-hand panels mark large non-acidic compartments with internal ILV puncta (Zoom). In right-hand panel, arrowhead marks ILV puncta (Zoom).

(**B**) SC also expressing RNAi targeting ESCRT-0 component, *Stam*. Number of non-acidic positive compartments with > 0.5 µm in diameter is increased, but very few contain ILVs (Zoom).

(**C**) SC also expressing RNAi targeting ESCRT-I component, *Vps28*. Number of non-acidic positive compartments with > 0.5 µm in diameter is increased, but very few contain ILVs (Zoom).

(**D**) SC also expressing RNAi targeting ESCRT-III component, *shrb*. Number of non-acidic positive compartments with > 0.5 µm in diameter is increased, but very few contain ILVs (Zoom).

(**E**) Bar chart showing the number of large (greater than 0.5 µm in diameter) non-acidic compartments marked by CD63-GFP in control and *ESCRT* knockdown SCs. Data from 39 SCs (three per gland) are shown.

(**F**) Bar chart showing the proportion of large (greater than 0.5 µm in diameter) non-acidic compartments containing CD63-GFP-positive ILVs in control and *ESCRT* knockdown SCs. Data from 39 SCs (three per gland) are shown.

(**G**) Bar chart showing the total number of CD63-GFP fluorescent puncta in three Z-planes from the lumen of AGs following *ESCRT* knockdown in SCs, compared to control SCs (n ≥ 10 AG lumens).

All images are from six-day-old male flies shifted to 29°C at eclosion. Genotypes are: *w; P[w$^+$, UAS-CD63-GFP] P[w$^+$, tub-GAL80$^{ts}$]/+*; *dsx-GAL4/+* with no knockdown construct (**A**), UAS-*Stam*-RNAi (HMS01429; **B**), UAS-*Vps28*-RNAi (v31894; **C**) or UAS-*shrb*-RNAi (v106823; **D**).

Scale bar in **A**-**D** (5 µm) and in **A**-**D** Zoom (1 µm). Data were analysed by one-way ANOVA. ****P < 0.0001, **P < 0.01, *P < 0.05.

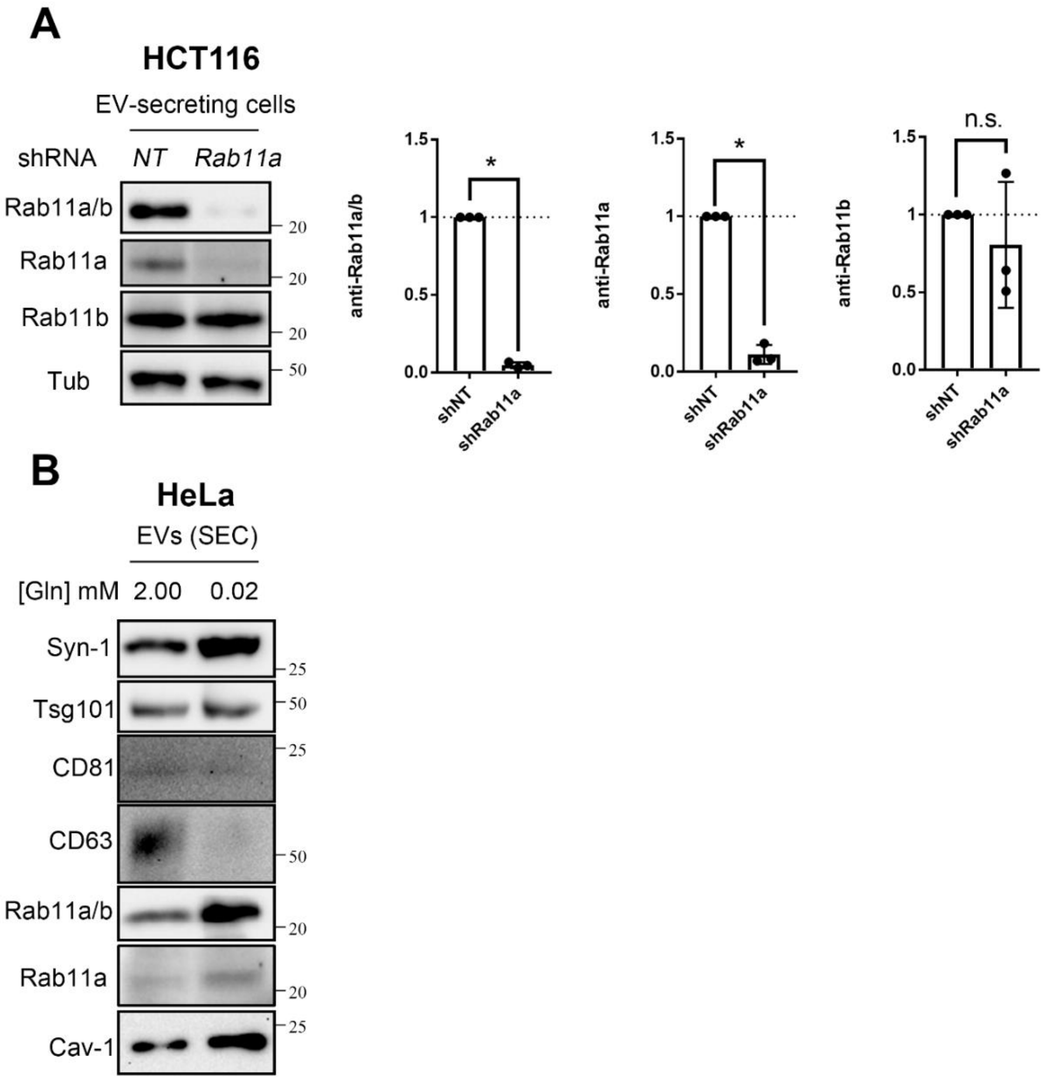


**Appendix Figure S3. Rab11a is the primary Rab11 isoform expressed in HCT116 cells**

(**A**) Western blot analysis of lysates from HCT116 cells with and without knockdown of *Rab11a*. Note that an antibody that specifically cross-reacts with Rab11a produces a much weaker signal than a specific Rab11b antibody. However, an antibody that can cross-react with both isoforms, primarily detects Rab11a, because the signal is strongly reduced after *Rab11a* knockdown, indicating that Rab11a is the predominant isoform in HCT116 cells, but that the Rab11a-specific antibody has a very low affinity for this isoform.

(**B**) Western blot analysis of EV preparations isolated by SEC from HeLa cells in glutamine-replete and -depleted conditions shows that Rab11a, identified by an isoform-specific antibody, is increased in EVs in the latter condition, mirroring the change seen with a non-isoform-specific antibody.

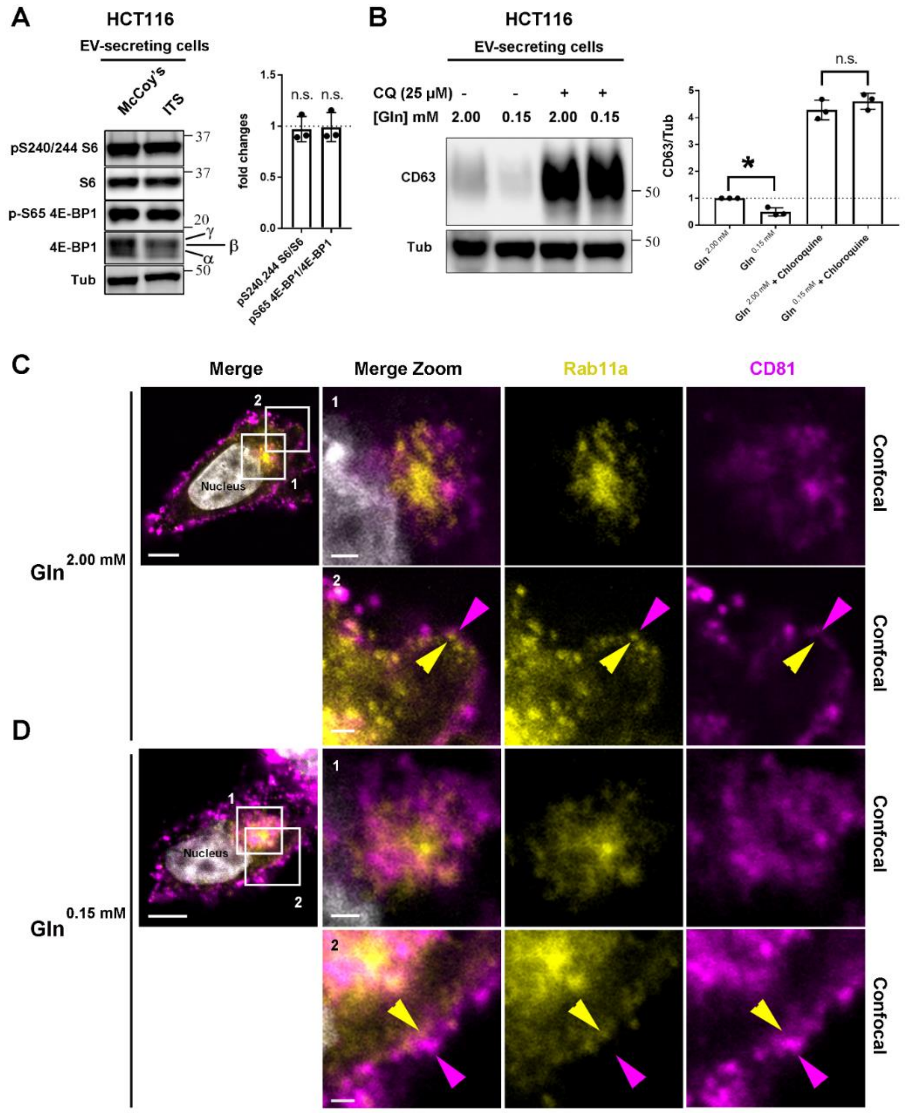


**Appendix Figure S4. Lysosomal turnover of CD63 and sub-plasma membrane localisation of Rab11a in HCT116 cells**

(**A**) Western blot analysis of lysates from HCT116 cells cultured for 24 h in McCoy's 5A modified medium supplemented with 10% FBS, or in serum-free basal medium (DMEM/F12) supplemented with 1% ITS. mTORC1 signalling was maintained under the latter conditions.

(**B**) Western blot analysis of lysates from HCT116 cells cultured for 24 h in glutamine-replete or -depleted conditions in the presence or absence of 25 µM chloroquine, which reduces acidification of lysosomes. Note that levels of CD63 are greatly increased in both culture conditions, but the difference in CD63 levels at different glutamine concentrations is lost.

Panels **C** and **D** show confocal images of fixed HCT116 cells, with boxed regions enlarged to the right. DAPI (grey) marks nucleus.

(**C**) In glutamine-replete conditions, there is limited co-localisation between Rab11a (yellow) and CD81 (magenta) in the perinuclear region containing clustered recycling endosomal compartments (1). Some CD81 is localised at the cell surface (magenta arrowhead), but the low levels of peripheral Rab11a are, as expected, primarily localised below the plasma membrane (yellow arrowhead), presumably in recycling endosomal compartments (2).

(**D**) Rab11a and CD81 are similarly localised under glutamine-depleted conditions.

Scale bar in Merge (5 µm), in Merge Zoom (1 µm). Bar charts derived from three independent experiments: *P < 0.05; n.s. = not significant.

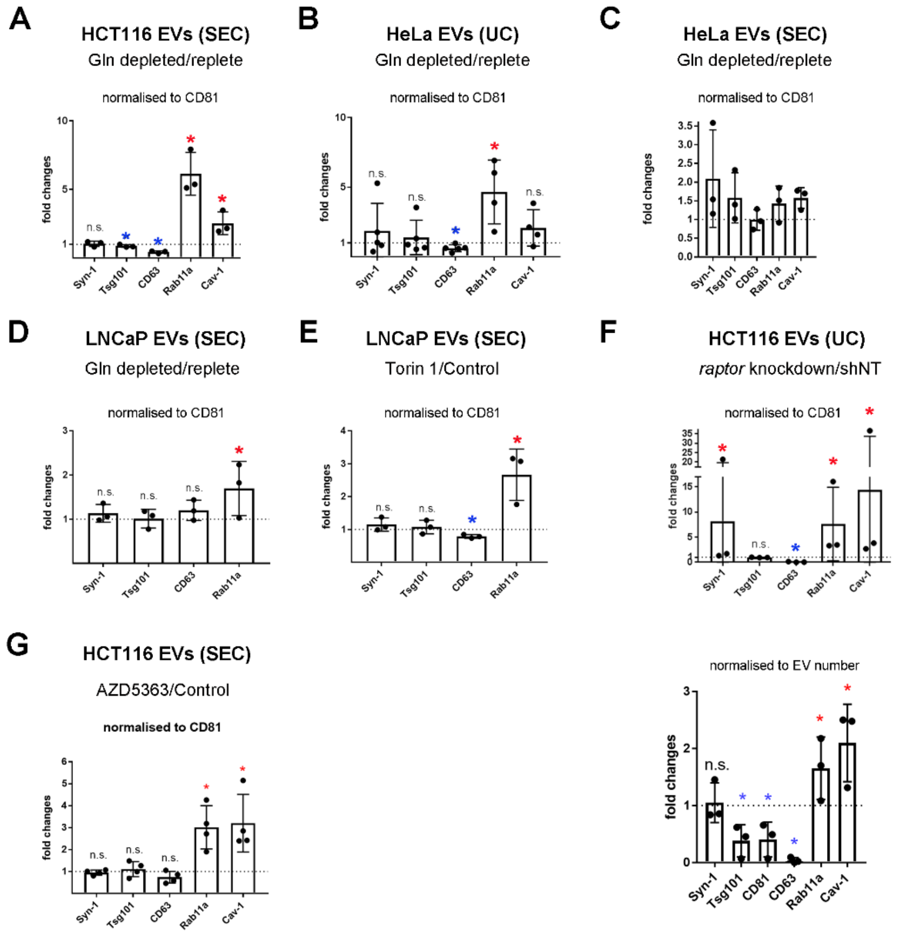


**H**

| EV Proteins | HCT116 [Gln]↓ | HCT116 *raptor* shRNA | HCT116 AZD5363 | HeLa [Gln]↓ | LNCaP [Gln]↓ | LNCaP Torin 1 |
|---|---|---|---|---|---|---|
| **Syn-1** | 1.32±0.22 * | 8.16±11.53 * | 1.02±0.16 | 1.86±1.98 | 1.13±0.20 | 1.15±0.20 |
| **Tsg101** | 0.96±0.18 | 0.96±0.04 | 1.10±0.30 | 1.39±1.24 | 1.01±0.21 | 1.07±0.21 |
| **CD63** | 0.33±0.32 * | 0.06±0.07 * | 0.81±0.27 | 0.62±0.25 ** | 1.20±0.23 | 0.79±0.06 * |
| **Rab11a** | 6.67±4.38 * | 7.59±7.29 * | 2.70±1.11 ** | 4.66±2.23 * | 1.7±0.61 * | 2.67±0.78 * |
| **Cav-1** | 4.58±2.03 * | 14.36±19.27 * | 2.96±1.26 ** | 2.08±1.31 | n.a. | n.a. |

**Appendix Figure S5. Glutamine depletion and Akt/mTORC1 signalling inhibition increase secretion of Rab11a relative to standard exosome markers**

Bar charts show changes in exosome markers relative to CD81 (**A**-**G**) and to EV number (**F**) in EV preparations from different cell lines subjected to a range of 'stress' conditions.

(**A**) Glutamine depletion of HCT116 cells using SEC for EV preparation.

(**B**) Glutamine depletion of HeLa cells using UC for EV preparation.

(**C**) Glutamine depletion of HeLa cells using SEC for EV preparation.

(**D**) Glutamine depletion of LNCaP cells using SEC for EV preparation.

(**E**) Torin1 inhibition mTORC1 in LNCaP cells using SEC for EV preparation.

(**F**) *raptor* knockdown in HCT116 cells using UC for EV preparation. Data are normalised both to CD81 and EV particle number.

(**G**) AZD5363 inhibition of Akt in HCT116 cells using SEC for EV preparation.

(**H**) Table summarising the EV protein analyses in Fig 5 (normalised to CD81 levels). Significantly decreased levels are in blue and increased levels are in red.

Bar charts derived from at least three independent experiments and analysed by the Kruskal-Wallis test: *P < 0.05; n.s. = not significant.

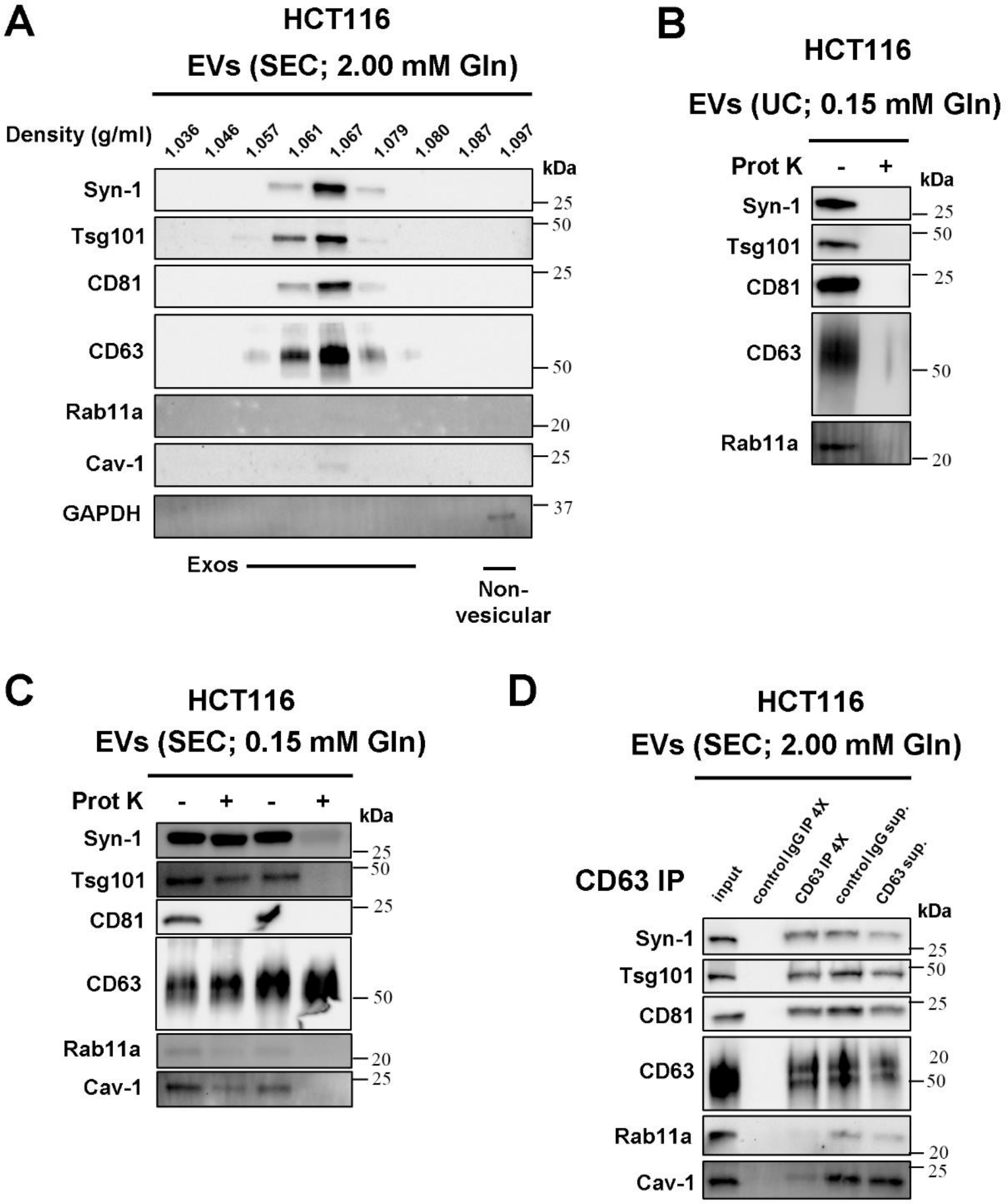


**Appendix Figure S6. EV-associated Rab11a is carried inside exosomes that are not marked by CD63**

Panels show western blot analyses of EV proteins from HCT116 cells.

(**A**) EV preparation from glutamine-replete HCT116 cells isolated by SEC and then separated by high-resolution iodixanol-PBS density centrifugation. Exosome markers fractionate at the expected densities, but because of the low levels of Rab11a, it is only detected weakly in the peak exosome fraction.

(**B**) EVs isolated by UC under glutamine-depleted conditions and then subjected to Proteinase K (Prot K) digestion in the presence of 1.1% LDS (Lithium dodecyl sulfate). All proteins are proteolytically degraded.

(**C**) EVs isolated by SEC under glutamine-depleted conditions and then subjected to Proteinase K (Prot K) digestion in the presence or absence of Triton® X-100 (Triton-X). Only membrane-associated CD81 is digested in the absence of detergent, while CD63 is resistant to digestion, even in its presence.

(**D**) EVs isolated under glutamine-replete conditions were immunocaptured with anti-CD63 antibodies coupled to magnetic beads. This method only pulls down a fraction of CD63, so the protein from pull-down of four times the input is loaded for comparison. Compared to other exosome proteins, low levels of Rab11a and Cav-1 are captured by anti-CD63 beads.

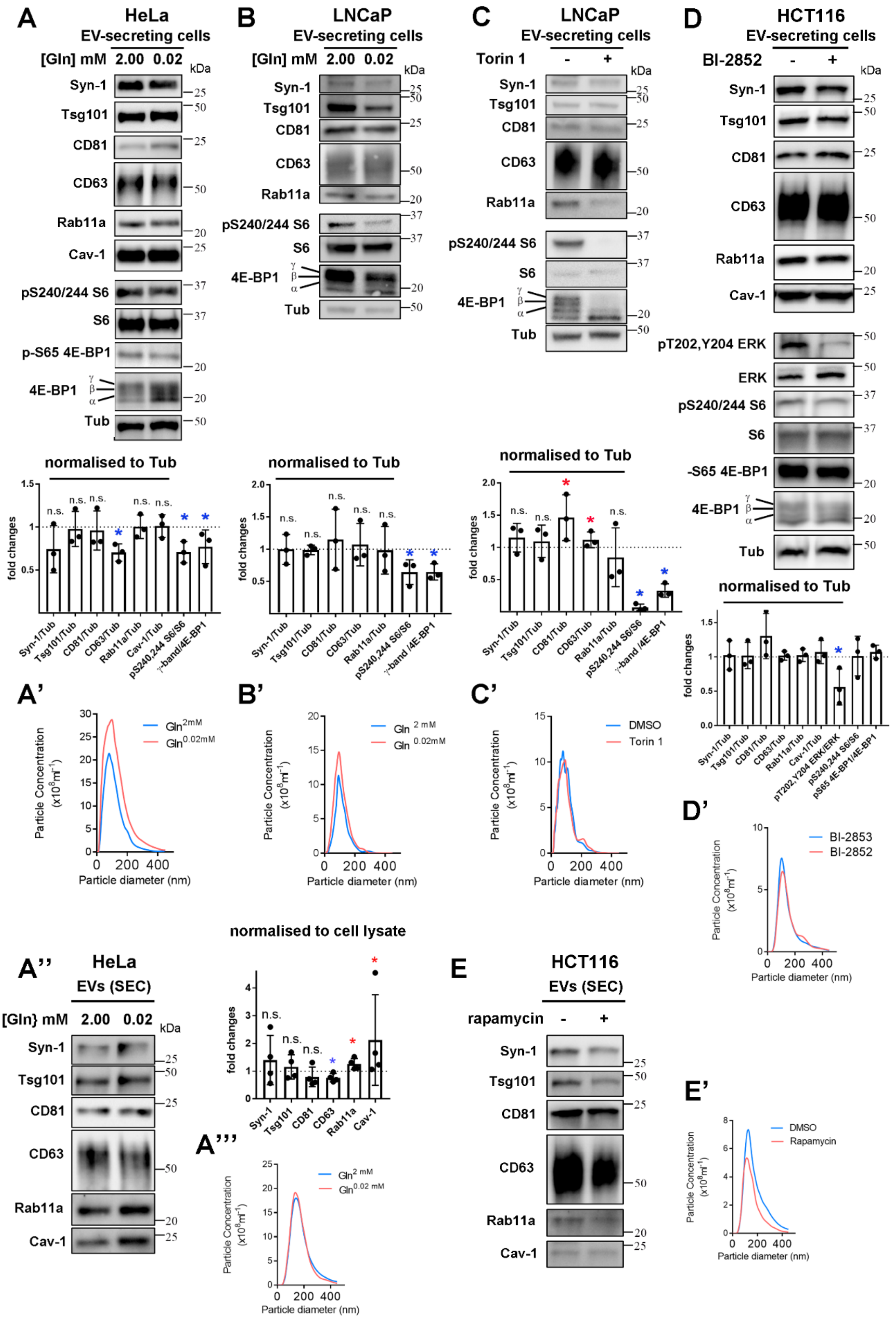
A
HeLa
EV-secreting cells
[Gln] mM 2.00 0.02
kDa
Syn-1
Tsg101
CD81
CD63
Rab11a
Cav-1
pS240/244 S6
S6
p-S65 4E-BP1
4E-BP1
Tub
normalised to Tub
fold changes
n.s.
Syn-1/Tub
Tsg101/Tub
CD81/Tub
CD63/Tub
Rab11a/Tub
Cav-1/Tub
pS240,244 S6/S6
γ-band/4E-BP1
B
LNCaP
EV-secreting cells
[Gln] mM 2.00 0.02
C
LNCaP
EV-secreting cells
Torin 1 - +
D
HCT116
EV-secreting cells
BI-2852 - +
pT202,Y204 ERK
ERK
-S65 4E-BP1
pT202,Y204 ERK/ERK
pS65 4E-BP1/4E-BP1
A'
B'
C'
D'
Particle Concentration (x10^8 ml^-1)
Particle diameter (nm)
Gln 2mM
Gln 0.02mM
DMSO
Torin 1
BI-2853
BI-2852
A''
HeLa
EVs (SEC)
[Gln} mM 2.00 0.02
normalised to cell lysate
A'''
E
HCT116
EVs (SEC)
rapamycin - +
E'
DMSO
Rapamycin

**Appendix Figure S7. Changes in EV proteins in cell lysates and EVs following glutamine depletion, reduction in PI3K/Akt/mTORC1 or KRAS signalling in human cancer cell lines**

Panels show western blot analyses of cell and EV proteins, as well as Nanosight Tracking Analysis (NTA) of EV size and number. Bar charts indicate abundance of putative exosome proteins relative to Tubulin in cell lysates. Gel loading for EV preparations was normalised to cell lysate protein levels.

(**A**) With relevance to EVs shown in Fig 5A, western blot analysis of lysates from HeLa cells cultured in glutamine-replete (2.00 mM) and -depleted (0.02 mM) medium for 24 h. Total protein levels were reduced by 19 ± 4% after glutamine depletion.

(**A'**) NTA for EV samples produced as in Fig 5A.

(**A''**) Western blot analysis of EVs isolated by SEC from HeLa cells cultured in glutamine-replete (2.00 mM) and -depleted (0.02 mM) medium for 24 h. Total protein levels were reduced by 19 ± 4% after glutamine depletion.

(**A'''**) NTA for EV samples produced as in Appendix Fig S7A''.

(**B**) With relevance to EVs shown in Fig 5B, western blot analysis of lysates from LNCaP cells cultured in glutamine-replete (2.00 mM) and -depleted (0.02 mM) media for 24 h. Total protein levels were unaffected (100 ± 11% of control values) after glutamine depletion.

(**B'**) NTA for EV samples produced as in Fig 5B.

(**C**) With relevance to EVs shown in Fig 5C, western blot analysis of lysates from LNCaP cells cultured in the presence or absence of 120 nM Torin 1 for 24 h. Total protein levels were reduced by 19 ± 1% following drug treatment.

(**C'**) NTA for EV samples produced as in Fig 5C.

(**D**) With relevance to EVs in Fig 5F, western blot analysis of lysates from HCT116 cells cultured in the presence of 10 µM KRAS inhibitor Bl-2852 or control compound Bl-2853 for 24 h.

(**D**') NTA for EV samples produced as in Fig 5F.

(**E**) EVs isolated by SEC of medium from HeLa cells cultured in glutamine-replete (2.00 mM) and -depleted (0.02 mM) conditions for 24 h.

(**E'**) NTA for EV samples produced as in **E**.

Bar charts derived from at least three independent experiments and analysed by the Kruskal-Wallis test:
*$P < 0.05$; n.s. = not significant.

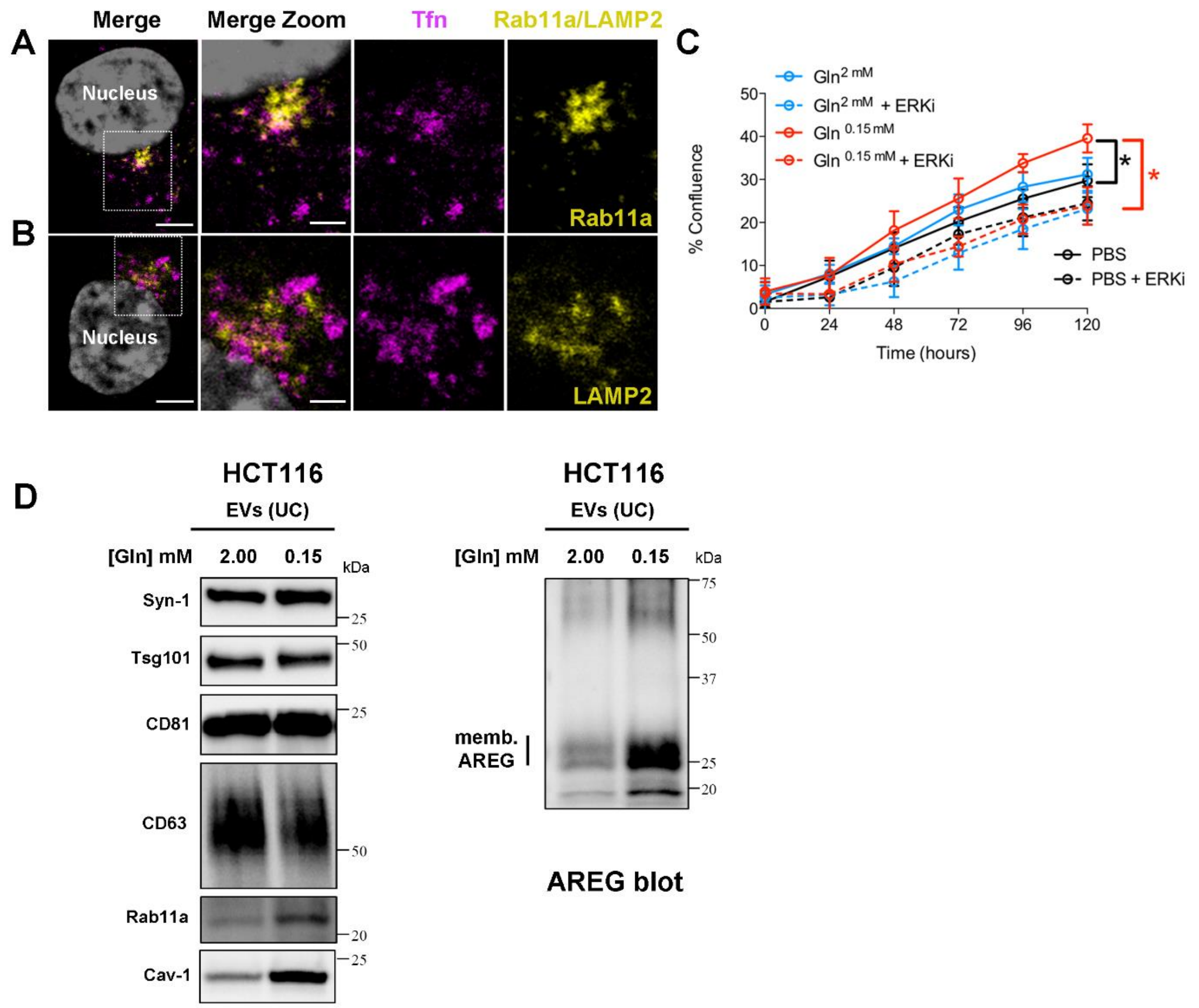


**Appendix Figure S8. Transferrin uptake, the effects of ERK inhibition on HCT116 recipient cell growth and AREG isoforms associated with HCT116 EVs**

(**A**) With relevance to the protein uptake assays shown in Fig 6D, HCT116 cells stained with an anti-Rab11a antibody (yellow), following uptake of fluorescent Alexa-488-conjugated Tfn (magenta); boxed region enlarged in Zoom. Some Tfn co-localises with Rab11a. DAPI (grey) stains nucleus.

(**B**) Cells stained with an anti-LAMP2 antibody (yellow), following uptake of fluorescent Alexa-488-conjugated Tfn (magenta); boxed region enlarged in Zoom. There is little overlap between Tfn and LAMP2. DAPI (grey) stains nucleus.

(**C**) With relevance to Fig 7B, growth curves for HCT116 recipient cells after pre-treatment with EV preparations isolated as in Fig 7A or with PBS, and then incubated for the first 24 h of culture in the presence or absence of the ERK inhibitor SCH772984 (1.00 µM).

Scale bars in **A**, **B** (5 µm) and in Zoom (2 µm). Growth curve was reproduced in three independent experiments and analysed by two-way ANOVA. *P colour denotes significant increase relative to EVs from glutamine-replete cells with no inhibitor (black) and to EVs from glutamine-depleted cells after recipient cells were treated with SCH772984 (red): *P < 0.05; n.s. = non-significant.

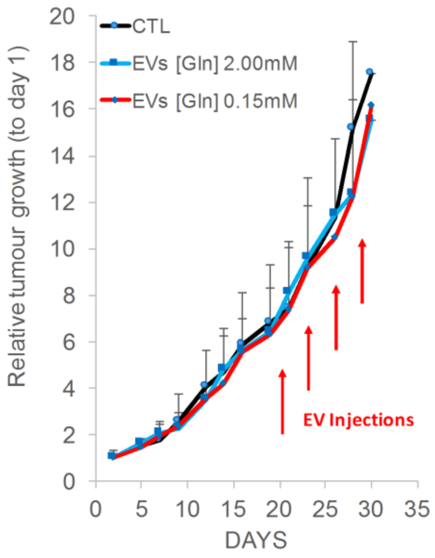


**Appendix Figure S9. Overall growth of HCT116 tumours in xenograft mouse model is not affected by EV injection over a nine-day period**

The size of HCT116 tumours grown in xenograft mouse models, generated and treated as in Fig 8, was measured every two to three days. Arrows mark times of the four EV injections in to the tumour. There was no significant difference in size for any of the treatments: PBS injection control, EVs isolated from glutamine-replete control or glutamine-depleted HCT116 cells.
