# Supplementary material for: Glutamine deprivation alters the origin and function of cancer cell exosomes
Source: EMBO J. 2020 Jul 28;39(16):e103009. doi: 10.15252/embj.2019103009 (PMC7429491; doi:10.15252/embj.2019103009)
Supplement: Supplementary file 2 — Expanded View Figures PDF [file EMBJ-39-e103009-s002.pdf]

# Expanded View Figures

**Figure EV1. Exosomes are made inside Rab11 compartments in *Drosophila* secondary cells.**

A Bar chart showing the number of ILVs within specific size ranges in the non-acidic compartments of SCs expressing CD63-GFP. Each dot represents number of vesicles within each size range in a single compartment. The size of all ILVs was measured from 3D-SIM images of at least three compartments for three SCs per gland from three different animals (producing at least 27 dots per size range).

B Bar chart shows average number of large (diameter greater than two micrometres), non-acidic dense-core granule compartments (identified using DIC microscopy) in SCs from $w^{1118}$, *YFP-Rab11* gene trap, *SC>Btl-GFP* and SC>*CD63-GFP* male flies. Note that the number of these compartments is increased by CD63-GFP expression, as previously reported (Redhai *et al*, 2016), but not by the other transgenes. Counts are for three SCs per gland from 10 different animals.

C Bar chart shows average number of large (diameter greater than two micrometres), LysoTracker Red®-positive, acidic compartments (LELs) in different genetic backgrounds. The number of acidic compartments is slightly increased by the *YFP-Rab11* gene trap and *SC > Btl-GFP*. Counts are for three SCs per gland from 10 different animals.

D Bar chart shows average size of the largest acidic LEL compartment in these genetic backgrounds. This is slightly reduced by the *YFP-Rab11* gene trap and increased by CD63-GFP. Sizes were measured for three SCs per gland from 10 different animals.

E Transmission electron micrograph of a $w^{1118}$ non-transgenic fly SC in the AG epithelium with boxes showing location of enlarged images, marked by coloured dots. In the magnified green dot image, most large compartments lacking dense-core granules are equivalent to the acidic compartments seen in live fluorescence imaging (Corrigan *et al*, 2014). Arrows mark exosome-sized (30–150 nm diameter) vesicles. Arrowheads mark complex membranous structures, characteristic of lysosomal compartments. In the magnified red and yellow dot images, large non-acidic, dense-core granule compartments have previously been reported to be Rab11-positive (Redhai *et al*, 2016). Arrows mark ILVs. Arrowhead in each image marks dense-core granule, which is surrounded by filamentous material (Corrigan *et al*, 2014).

F–K Wide-field fluorescence images of living fly secondary cells (SCs) and paired accessory glands (AGs; K), and confocal images of fixed AG lumens (G–I). Focal planes indicated in Fig 1A schematics. Acidic compartments are marked by the vital dye LysoTracker® Red (magenta); GFP- and YFP-tagged constructs are shown in yellow. (F) Basal view of an SC expressing a *YFP-Rab11* gene trap, with cell outline approximated by dashed white circle. Rab11 compartment highlighted by box contains three large ILVs (arrowhead) with Rab11 marking their outlines. (G) Transverse view of an SC from a fly in which CD63-GFP is expressed specifically in SCs under GAL4/UAS control. CD63-GFP is present on both the limiting and ILV membranes of acidic and non-acidic compartments, in addition to the apical plasma membrane (arrows in merge). Image is also shown schematically. CD63-GFP puncta (arrows) are present in the AG lumen of these flies. (H) Transverse view of an SC expressing a *YFP-Rab11* gene trap, which marks non-acidic compartments, and also the cytosol, but is not trafficked to the apical plasma membrane (arrows in merge). Image is also shown schematically. Absence of YFP-Rab11 throughout the apical plasma membrane is shown in the Z-stack in Movie EV2. YFP-Rab11 puncta (arrows) are present at low levels in the AG lumen. (I) Basal view of an SC from a fly expressing SC-specific YFP-Rab11 (yellow) under GAL4/UAS control, with cell outline approximated by dashed white circle. Boxed non-acidic compartment is magnified in Zoom; arrows highlight YFP-Rab11-positive ILVs (Merge) and puncta present at low levels (AG lumen). (J) Basal view of an SC from a fly expressing the *YFP-Rab11* gene trap (yellow) and SC-specific CD63-mCherry (magenta) under GAL4/UAS control, with cell outline approximated by dashed white circle. Large non-acidic compartments with CD63-mCherry at their limiting membrane appear to exclude YFP-Rab11 from their surface (boxes 1 and 2). In the compartment lumen, co-localisation of the markers is observed (white arrowheads; box 1), but also non-overlapping fluorescence (coloured arrowheads; box 2), consistent with ILV heterogeneity in a single compartment. (K) AGs from a fly expressing nuclear GFP under the control of a well-characterised *btl-GAL4* enhancer trap (Hayashi *et al*, 2002). Boxed region of AG epithelium containing SCs is enlarged in Zoom, with SC outlines approximated by dashed white circles. GFP localisation in binucleate SCs suggests that *btl* is normally expressed in SCs.

Data information: All images and data are from 6-day-old males shifted to 29°C at eclosion. Genotypes of flies carrying multiple transgenes are as follows: *w; P[$w^+$, UAS-CD63-GFP] P[$w^+$, tub-GAL80$^{ts}$]/+; dsx-GAL4/+* (A–D, G), *w; P[$w^+$, tub-GAL80$^{ts}$]/+; dsx-GAL4/P[$w^+$, UAS-btl-GFP]* (B–D); *w; P[$w^+$, tub-GAL80$^{ts}$]/+; dsx-GAL4/P[$w^+$, UAS-YFP-Rab11]* (I), *w; P[$w^+$, tub-GAL80$^{ts}$]/+; dsx-GAL4, TI{TI}Rab11$^{EYFP}$/P[$w^+$, UAS-CD63-mCherry]* (J); *w; P[$w^+$, UAS-GFP$^{nls}$]/+; btl$^{NP6593}$/+* (K). Scale bar in G (5 μm) applies to G-J, and in AG lumen in G (20 μm) applies to G-I. Other scale bars are as follows: 5 μm (E), 1 μm (E, green and red dots), 0.5 μm (E, yellow dot), 5 μm (F, J), 1 μm (F and J, Zoom), 2 μm (I, Zoom), 250 μm (K), 20 μm (K, Zoom). For A-D, values for transgenic flies are compared to $w^{1118}$, using one-way ANOVA. ****$P < 0.0001$; ***$P < 0.001$; **$P < 0.01$; ns = not significant. Bars and error bars denote mean ± SD.

 © 2020 The Authors

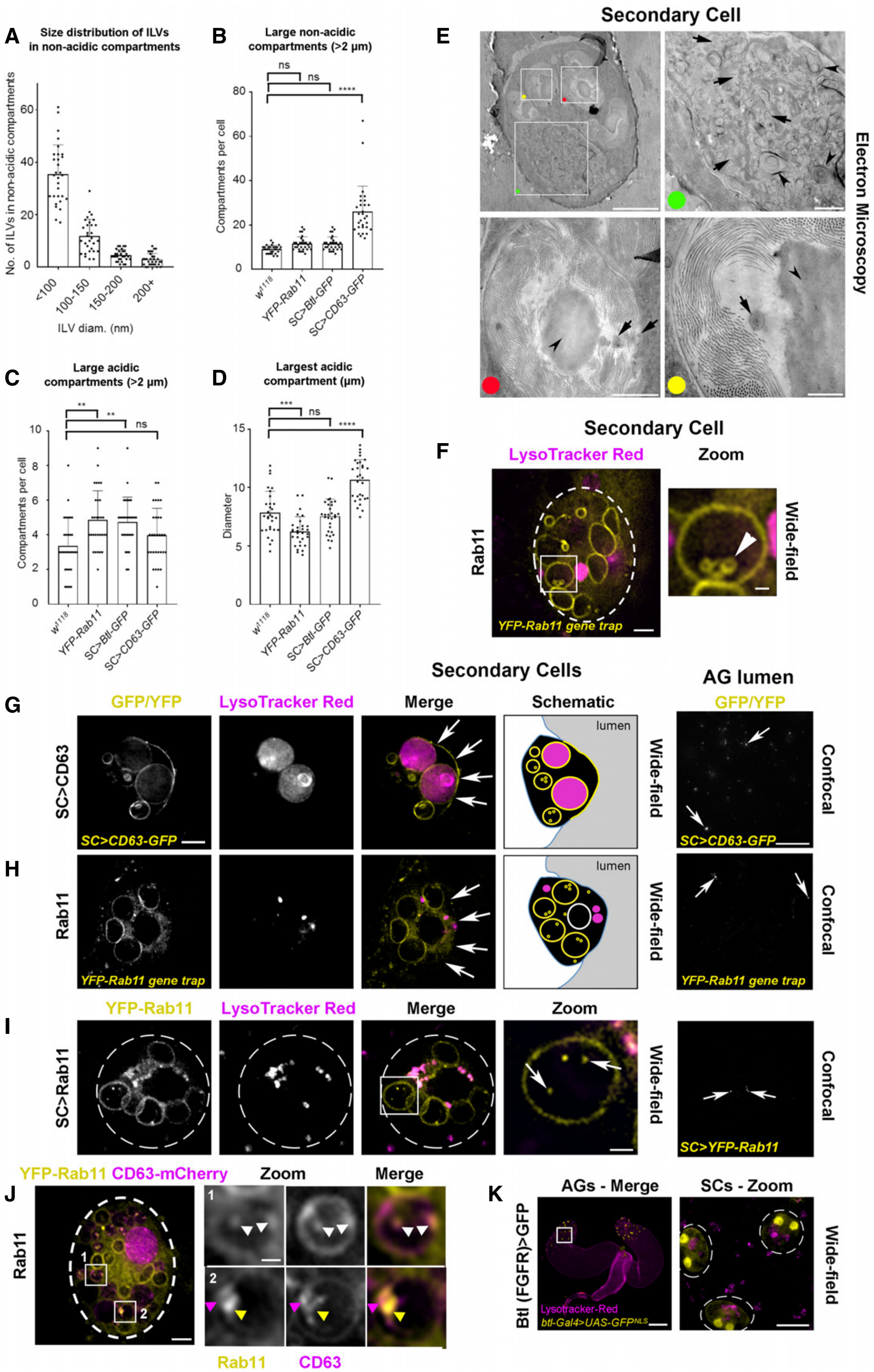
A
Size distribution of ILVs in non-acidic compartments
No. of ILVs in non-acidic compartments
ILV diam. (nm)
<100
100-150
150-200
200+
B
Large non-acidic compartments (>2 μm)
Compartments per cell
ns
****
C
Large acidic compartments (>2 μm)
Compartments per cell
D
Largest acidic compartment (μm)
Diameter
***
$w^{1118}$
YFP-Rab11
SC>Btl-GFP
SC>CD63-GFP
E
Secondary Cell
Electron Microscopy
F
Secondary Cell
LysoTracker Red
Zoom
Rab11
YFP-Rab11 gene trap
Wide-field
Secondary Cells
AG lumen
G
GFP/YFP
LysoTracker Red
Merge
Schematic
lumen
SC>CD63
SC>CD63-GFP
Wide-field
Confocal
H
Rab11
YFP-Rab11 gene trap
I
YFP-Rab11
Zoom
SC>Rab11
SC>YFP-Rab11
J
YFP-Rab11 CD63-mCherry
Rab11
CD63
K
AGs - Merge
SCs - Zoom
Btl (FGFR)>GFP
Lysotracker-Red
btl-Gal4>UAS-GFP^NLS

**Figure EV1.**

**Figure EV2. ESCRTs regulate Rab11-labelled exosome biogenesis in the Rab11-positive compartments of *Drosophila* secondary cells.**

A–D Basal wide-field fluorescence views through living secondary cells (SCs) expressing the *YFP-Rab11* gene trap, with cell outline approximated by dashed white circle. Acidic compartments are marked with LysoTracker Red® (magenta). Boxed non-acidic compartment in Merge is magnified in Zoom. (A) SC with no RNAi expressed (control). Arrowheads in left-hand panels mark YFP-Rab11-positive compartments with internal ILV puncta. In right-hand panel, arrowhead marks intraluminal puncta (Zoom). (B) SC also expressing RNAi targeting ESCRT-0 component, *Stam*. Number of Rab11-positive compartments is unchanged, but few contain ILV puncta (arrowheads and specific example highlighted in Zoom). (C) SC also expressing RNAi targeting ESCRT-I component, *Vps28*. Number of small Rab11-positive compartments is increased, but few are > 1 μm in diameter and virtually none contain ILVs (Zoom). (D) SC also expressing RNAi targeting ESCRT-III component *shrb*. Number of small Rab11-positive compartments is increased, but few are > 1 μm in diameter and virtually none contain ILVs (Zoom).

E Bar chart showing the number of large (greater than one micrometre in diameter) non-acidic compartments marked by YFP-Rab11 in control and *ESCRT* knockdown SCs. Data from 30 SCs (three per gland; $n = 16$ for *Stam*-RNAi; most values are zero for *Vps28* and *shrb* knockdown) are shown.

F Bar chart showing the proportion of large (greater than one micrometre in diameter) non-acidic compartments containing YFP-Rab11-positive ILV puncta in control and *ESCRT* knockdown SCs. Data from 30 SCs (three per gland; $n = 16$ for *Stam*-RNAi; most values are zero for *Vps28* and *shrb* knockdown) are shown.

G Basal wide-field fluorescence view through living secondary cell (SC) after a 4-h pulse of Shrb-GFP (yellow) expression, with cell outline approximated by dashed white circle. Acidic compartments are marked with LysoTracker Red® (magenta). Boxed non-acidic compartment in Merge is magnified in Zoom. Arrows highlight Shrb-GFP localisation on the limiting membranes of large non-acidic compartments (Zoom). Complete view of Z-stack is shown in Movie EV3. Nuclear staining (marked "n") of these binucleate cells is non-specific.

Data information: All images are from 6-day-old male flies shifted to 29°C at eclosion, except for G, where flies were cultured at 25°C for 6 days before 29°C pulse. Genotypes are as follows: *ω; P[ω$^{+}$, tub-GAL80$^{ts}$]/+; dsx-GAL4 TI{TI}Rab11$^{EYFP}$/+* with no knockdown construct (A), UAS-*Stam*-RNAi (HMS01429; B), UAS-*Vps28*-RNAi (v31894; C) or UAS-*shrb*-RNAi (v106823; D). Genotype of male in G is *ω; P[ω+, UAS-shrb-GFP]/P[ω$^{+}$, tub-GAL80$^{ts}$]; dsx-GAL4/+*. Scale bar in A–D and G (5 μm) and in A–D and G Zoom (1 μm). Data were analysed by the Kruskal–Wallis test (E) and one-way ANOVA (F). ****$P < 0.0001$, ***$P < 0.001$, n.s. = not significant. Bars and error bars in panels E and F denote mean ± SD.

 © 2020 The Authors

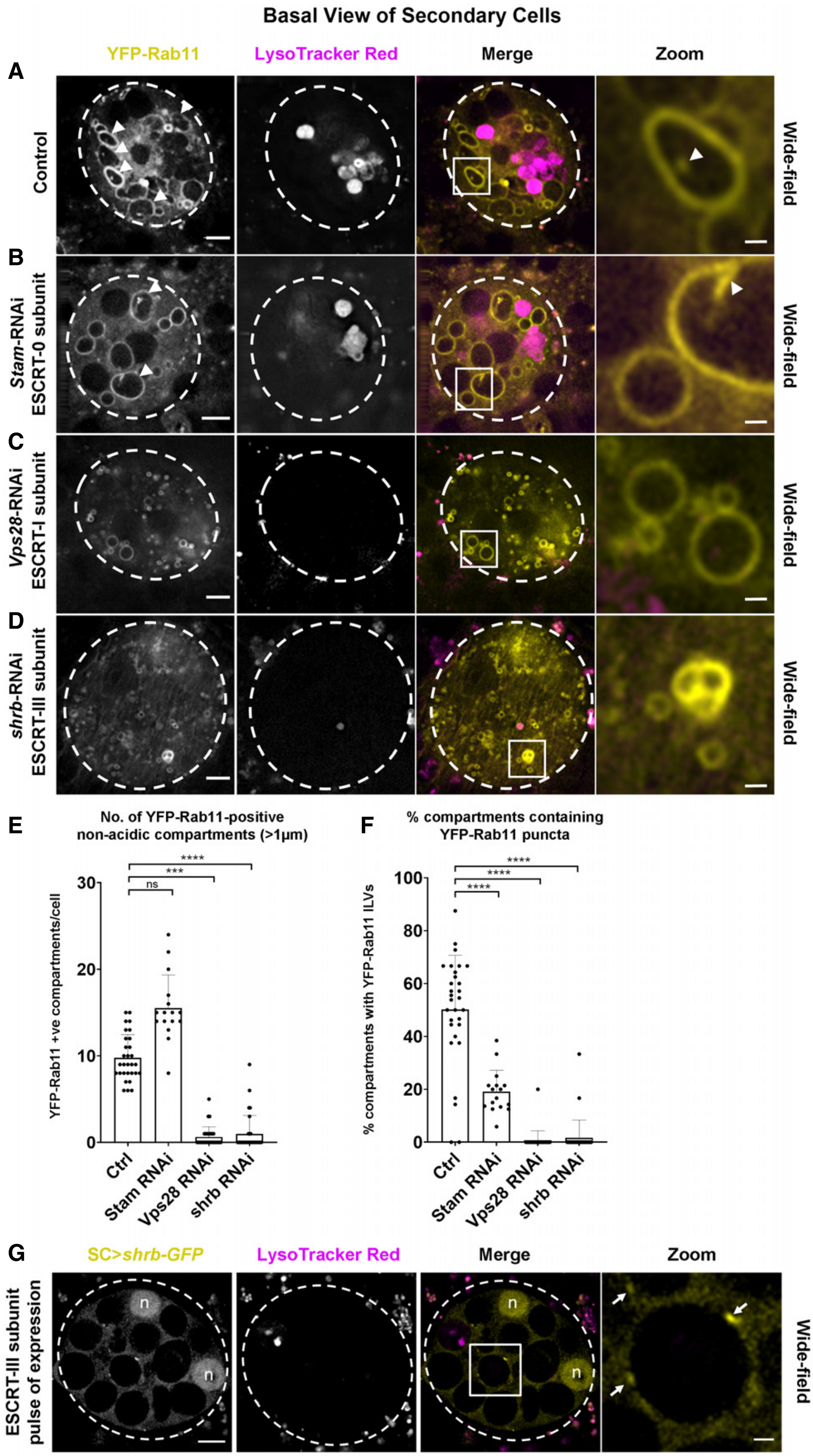
Basal View of Secondary Cells
A B C D
YFP-Rab11
LysoTracker Red
Merge
Zoom
Control
Stam-RNAi ESCRT-0 subunit
Vps28-RNAi ESCRT-I subunit
shrb-RNAi ESCRT-III subunit
Wide-field
E
No. of YFP-Rab11-positive non-acidic compartments (>1µm)
YFP-Rab11 +ve compartments/cell
ns
***
****
Ctrl
Stam RNAi
Vps28 RNAi
shrb RNAi
F
% compartments containing YFP-Rab11 puncta
% compartments with YFP-Rab11 ILVs
G
SC>shrb-GFP
ESCRT-III subunit pulse of expression
n

**Figure EV2.**

**Figure EV3. Effects of glutamine depletion on HCT116 cells and their EVs.**

Panels show the effect of different treatments on HCT116 colorectal cancer cells and on the EVs they secrete.

A Bar chart showing relative levels of phosphorylation of mTORC1 downstream readouts, S6 and 4E-BP1 (measured as a ratio of p-S65-4E-BP1 to pan 4E-BP1 or of the γ-phosphorylated form of 4E-BP1 relative to total 4E-BP1), following growth of cells for 24 h in glutamine-depleted (2.00 mM) versus glutamine-replete (0.15 mM) conditions. Data are from triplicate experiments, analysed as in Fig 4A.

B Nanosight Tracking Analysis of EV size and number for diluted samples (normalised to cell lysate protein levels) produced from ultracentrifugation (UC) of medium from cells cultured in glutamine-replete and glutamine-depleted conditions for 24 h, as in Fig 4B.

C Electron micrographs (EMs) of EV samples from glutamine-replete and glutamine-depleted cells as analysed in (B). Arrows indicate representative EVs with the characteristic cup-shaped morphology previously reported. Scale bars represent 500 nm (left panels) and 200 nm (right panels).

D Comparative Western analysis of putative exosome and non-exosome markers in HCT116 cell lysate (cells) and EV preparation produced by UC from cells grown in glutamine-replete conditions (EVs). Lanes are loaded with equal amounts of protein. Note that unlike other "classical" exosome markers, Rab11a and Cav-1 are present, but not enriched in EVs, while Golgi (TGN46), ER (calnexin) and early endosome (EEA1) markers are absent.

E Bar charts show changes in levels of putative exosome proteins in EVs produced by UC from cells grown in glutamine-depleted versus glutamine-replete conditions and analysed as in Fig 4B. Protein quantities in each sample were normalised to EV particle number, CD81, Syn-1 and Tsg101 in the four graphs ($n$ = 4, except for particle number, where $n$ = 6). All show an increase in Rab11a and Cav-1, and a decrease in CD63, while small or no change is seen for other markers.

F Western blot analysis of EV preparations isolated from HCT116 cells cultured in glutamine-replete and glutamine-depleted conditions for 24 h using size-exclusion chromatography (SEC; fractions two to four). EV loading was normalised to protein levels in cell lysates. Bar chart shows relative levels of putative exosome markers normalised to cell lysate protein mass ($n$ = 3). (F′) Nanosight Tracking Analysis of EV size and number for samples produced as in (F).

Data information: Bar charts derived from at least three independent experiments and analysed by the Kruskal–Wallis test; *$P < 0.05$; n.s. = not significant. Bars and error bars denote mean ± SD. Significantly decreased levels are in blue and increased levels are in red.


© 2020 The Authors

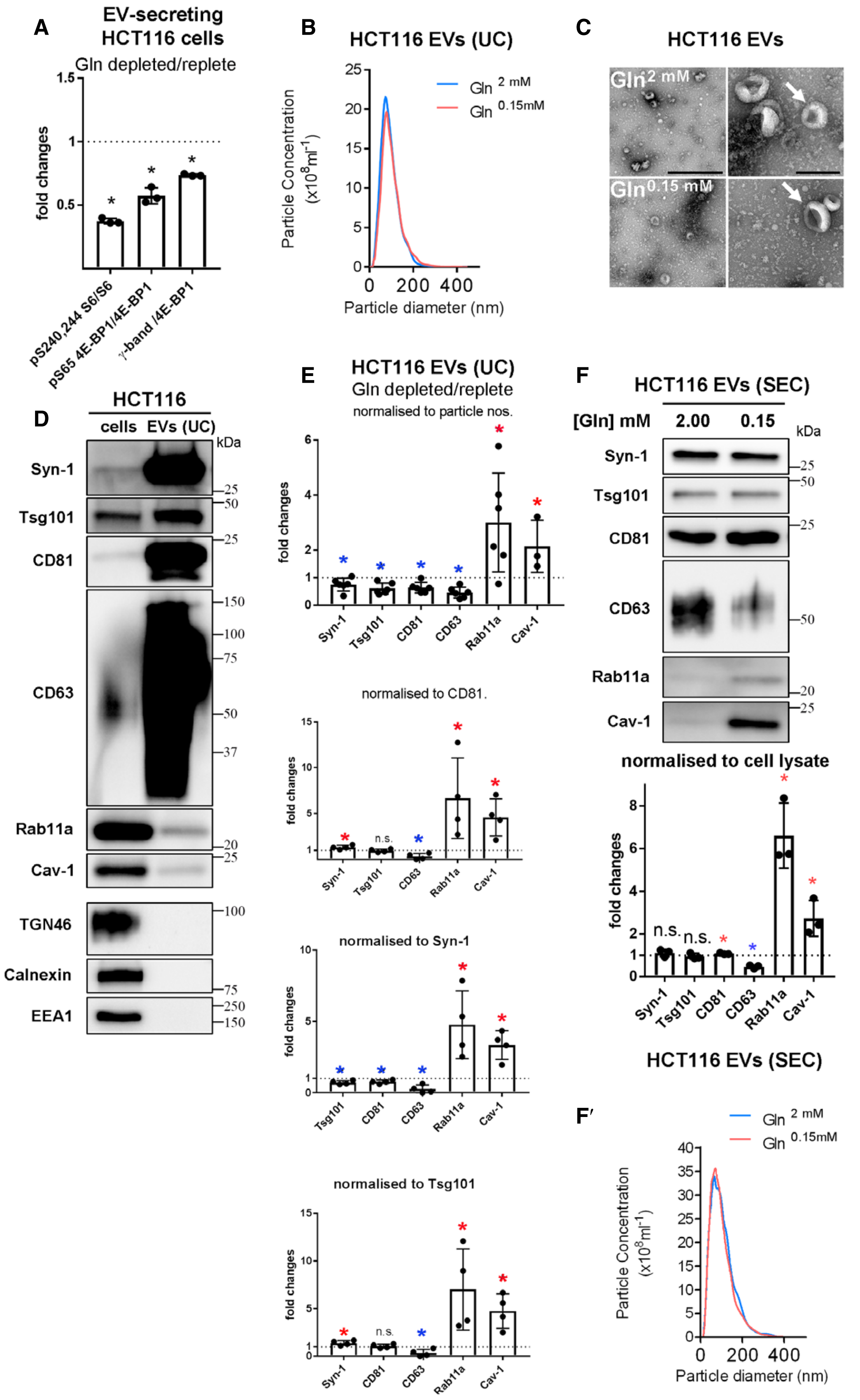
A
EV-secreting HCT116 cells
Gln depleted/replete
fold changes
pS240,244 S6/S6
pS65 4E-BP1/4E-BP1
γ-band /4E-BP1
B
HCT116 EVs (UC)
Particle Concentration (x10⁸ml⁻¹)
Particle diameter (nm)
Gln 2 mM
Gln 0.15mM
C
HCT116 EVs
Gln 2 mM
Gln 0.15 mM
D
HCT116
cells EVs (UC)
kDa
Syn-1
Tsg101
CD81
CD63
Rab11a
Cav-1
TGN46
Calnexin
EEA1
E
HCT116 EVs (UC)
Gln depleted/replete
normalised to particle nos.
normalised to CD81.
normalised to Syn-1
normalised to Tsg101
F
HCT116 EVs (SEC)
[Gln] mM
2.00
0.15
normalised to cell lysate
F′
HCT116 EVs (SEC)

Figure EV3.

**Figure EV4. Changes in EV proteins in cell lysates and EVs following reduction in PI3K/Akt/mTORC1 signalling in HCT116 cells.**

Panels show Western blot analyses of cell and EV proteins, as well as Nanosight Tracking Analysis (NTA) of EV size and number.

A Western blot analysis of lysates from HCT116 cells cultured in the presence or absence of 100 nM Torin 1 for 24 h. Total protein levels were reduced by approximately 20% following drug treatment. Bar chart shows the abundance of putative exosome proteins relative to tubulin in these lysates and relative levels of phosphorylation of mTORC1 downstream readouts, S6 and 4E-BP1 (measured as a ratio of p-S65-4E-BP1 to pan 4E-BP1). Significantly decreased levels are in blue and increased levels are in red. (A′) Western blot analysis of EV preparations isolated by ultracentrifugation from HCT116 cells cultured in the presence or absence of 100 nM Torin 1 for 24 h. EV loading was normalised to protein level in cell lysates. (A″) NTA for EV samples produced as in (A′).

B Western blot analysis of lysates from HCT116 cells cultured in the presence or absence of 10 nM rapamycin for 24 h. Total protein levels were reduced by 7 ± 2% following drug treatment. Bar chart shows the abundance of putative exosome proteins relative to tubulin in these lysates and relative levels of phosphorylation of mTORC1 downstream readouts, S6 and 4E-BP1 (measured as a ratio of p-S65-4E-BP1 to pan 4E-BP1). Significantly decreased levels are in blue and increased levels are in red. (B′) Western blot analysis of EV preparations from HCT116 cells isolated by ultracentrifugation from HCT116 cells cultured in the presence or absence of 10 nM rapamycin for 24 h. EV loading was normalised to protein level in cell lysates. See Appendix Fig S7E for analysis of SEC-isolated EVs under these conditions. (B″) NTA for EV samples produced as in (B′).

C With relevance to EVs shown in Fig 5D, Western blot analysis of lysates from HCT116 cells subjected to 4 days of *raptor* or non-targeting (*NT*) shRNA knockdown. Total protein levels were not significantly altered by knockdown versus control. Bar chart shows the abundance of putative exosome proteins relative to tubulin in these lysates and relative levels of phosphorylation of mTORC1 downstream readouts, S6 and 4E-BP1 (measured as a ratio of p-S65-4E-BP1 to pan 4E-BP1). Significantly decreased levels are in blue and increased levels are in red. (C′) NTA for EV samples produced as in Fig 5D.

D With relevance to EVs shown in Fig 5E, Western blot analysis of lysates from HCT116 cells cultured in the presence or absence of 3 μM AZD5363 for 24 h. Total protein levels were reduced by 12 ± 5% following drug treatment. Note that phosphorylation of PRAS40, an Akt target, is reduced by drug treatment. Bar chart shows the abundance of putative exosome proteins relative to tubulin in these lysates and relative levels of phosphorylation of mTORC1 downstream readouts, S6 and 4E-BP1 (measured as a ratio of p-S65-4E-BP1 to pan 4E-BP1). Significantly decreased levels are in blue and increased levels are in red. (D′) NTA for EV samples produced as in Fig 5E.

E Table summarising relative EV secretion, and relative activity of mTORC1 assessed by analysing levels of 4E-BP1 and S6 phosphorylation under conditions shown in panels A–D, in Fig 4A and in Appendix Fig S7. Note strong inhibition of S6 phosphorylation in Torin 1- and rapamycin-treated HCT116 cells, which is associated with low levels of EV- and exosome-associated proteins and low EV particle number counts by NTA. Data analysed by the Kruskal–Wallis test: $*P < 0.05$. Significantly decreased levels versus control are in blue and increased levels are in red.

Data information: Bar charts derived from three independent experiments and analysed by the Kruskal–Wallis test: $*P < 0.05$; n.s. = not significant. Bars and error bars denote mean ± SD.


© 2020 The Authors

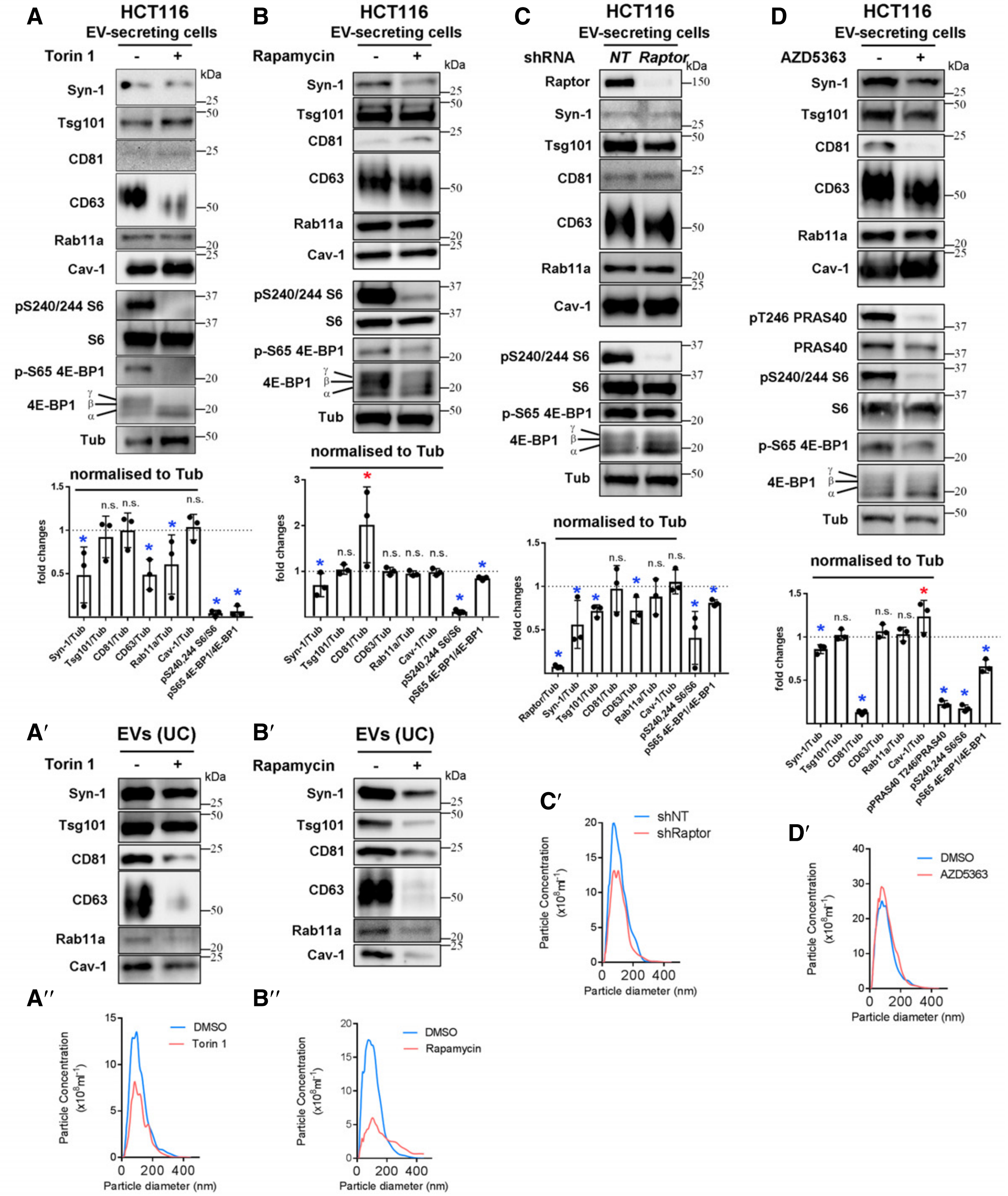


E

| mTORC1 activation | HCT116 [Gln]↓ | HCT116 Torin 1 | HCT116 Rapamycin | HCT116 *raptor* shRNA | HCT116 AZD5363 | HeLa [Gln]↓ | LNCaP [Gln]↓ | LNCaP Torin 1 |
|---|---|---|---|---|---|---|---|---|
| pS240,244 S6/ S6 | 0.38±0.02 * | 0.05±0.03 * | 0.12±0.04 * | 0.41±0.30 * | 0.18±0.04 * | 0.71±0.12 * | 0.64±0.19 * | 0.07±0.05 * |
| pS65 4EBP1 /4E-BP1 | 0.57±0.06 * | 0.07±0.06 * | 0.85±0.04 * | 0.81±0.04 * | 0.66±0.08 * | 0.92±0.26 | n.d. | n.d. |
| γ-band 4E-BP1 /4E-BP1 | 0.76±0.01 * | 0.07±0.03 * | 0.90±0.02 * | 0.86±0.08 * | 0.64±0.21 * | 0.77±0.20 * | 0.65±0.13 * | 0.33±0.10 * |
| EV numbers | 0.89±0.28 | 0.53 | 0.50 | 0.54±0.12 * | 1.16±0.25 | 1.43±0.36 | 1.01±0.08 | 1.20±0.07 * |

**Figure EV4.**

**Figure EV5. Analysis of EV function and proteins secreted by control and knockdown HCT116 colorectal cancer cells.**

A Number of DAPI-stained cells after 120-h culture in serum-depleted conditions (1% serum), following pre-incubation either with EVs from HCT116 cells cultured in glutamine-replete or glutamine-depleted conditions for 24 h, or with vehicle (PBS). Bar charts derived from three independent experiments.

B Overall levels of apoptosis, measured in arbitrary units (AU), of fluorescence, and levels of apoptosis per cell (right-hand graph) detected in HCT116 cells by analysis of caspase-3 and caspase-7 activities, following treatments with EVs isolated as in (A) or with vehicle (PBS).

C Growth curves for HCT116 recipient cells in 1% serum conditions following 30-min pre-incubation with $0.5 \times 10^3$, $1.0 \times 10^3$ and $2.0 \times 10^3$ EVs per cell, isolated by SEC from glutamine-replete and glutamine-depleted HCT116 cells or PBS.

D–F Western blot analysis of cell lysate proteins, and Nanosight Tracking Analysis (NTA) of EV size and number in D′ and E′. Bar charts indicate putative exosome protein levels normalised to Tubulin on Western blots. (D) With relevance to EVs shown in Fig 6C, Western blot analysis of lysates from EV-secreting cells with and without knockdown of *Rab11a* in glutamine-replete and glutamine-depleted conditions. Total protein levels were increased by $1 \pm 3\%$ following knockdown. Bar chart shows the abundance of putative exosome proteins relative to tubulin in these lysates and relative levels of phosphorylation of mTORC1 downstream readouts, S6 and 4E-BP1 (measured as a ratio of p-S65-4E-BP1 to pan 4E-BP1). Significantly decreased levels are in blue and increased levels are in red.(D′) NTA of EVs isolated from HCT116 cells with and without knockdown of *Rab11a*. (E) With relevance to EVs shown in Fig 6F, Western blot analysis of proteins isolated from EV-secreting HCT116 cells with and without knockdown of *Rab7*. Bar chart shows the abundance of putative exosome proteins relative to tubulin in these lysates and relative levels of phosphorylation of mTORC1 downstream readouts, S6 and 4E-BP1 (measured as a ratio of p-S65-4E-BP1 to pan 4E-BP1). Significantly decreased levels are in blue and increased levels are in red. (E′) NTA of EVs isolated from HCT116 cells with and without knockdown of *Rab7*. (F) With relevance to EVs shown in Fig 7C, Western blot analysis of cell lysates from HCT116 cells cultured in glutamine-replete (2.00 mM) and glutamine-depleted (0.15 mM) medium for 24 h. Gel was loaded with equal protein amounts. The activity of mTORC1 was assessed via phosphorylation of S6 and 4E-BP1. Bar chart shows the abundance of selected exosome/EV proteins relative to tubulin in these lysates. Significantly decreased levels are in blue and increased levels are in red.

Data information: Growth curves were reproduced in three independent experiments and analysed by two-way ANOVA. Bar charts derived from three independent experiments and analysed by the Kruskal–Wallis test: $^{**}P < 0.01$, $^{*}P < 0.05$ Bars and error bars denote mean ± SD.

 © 2020 The Authors

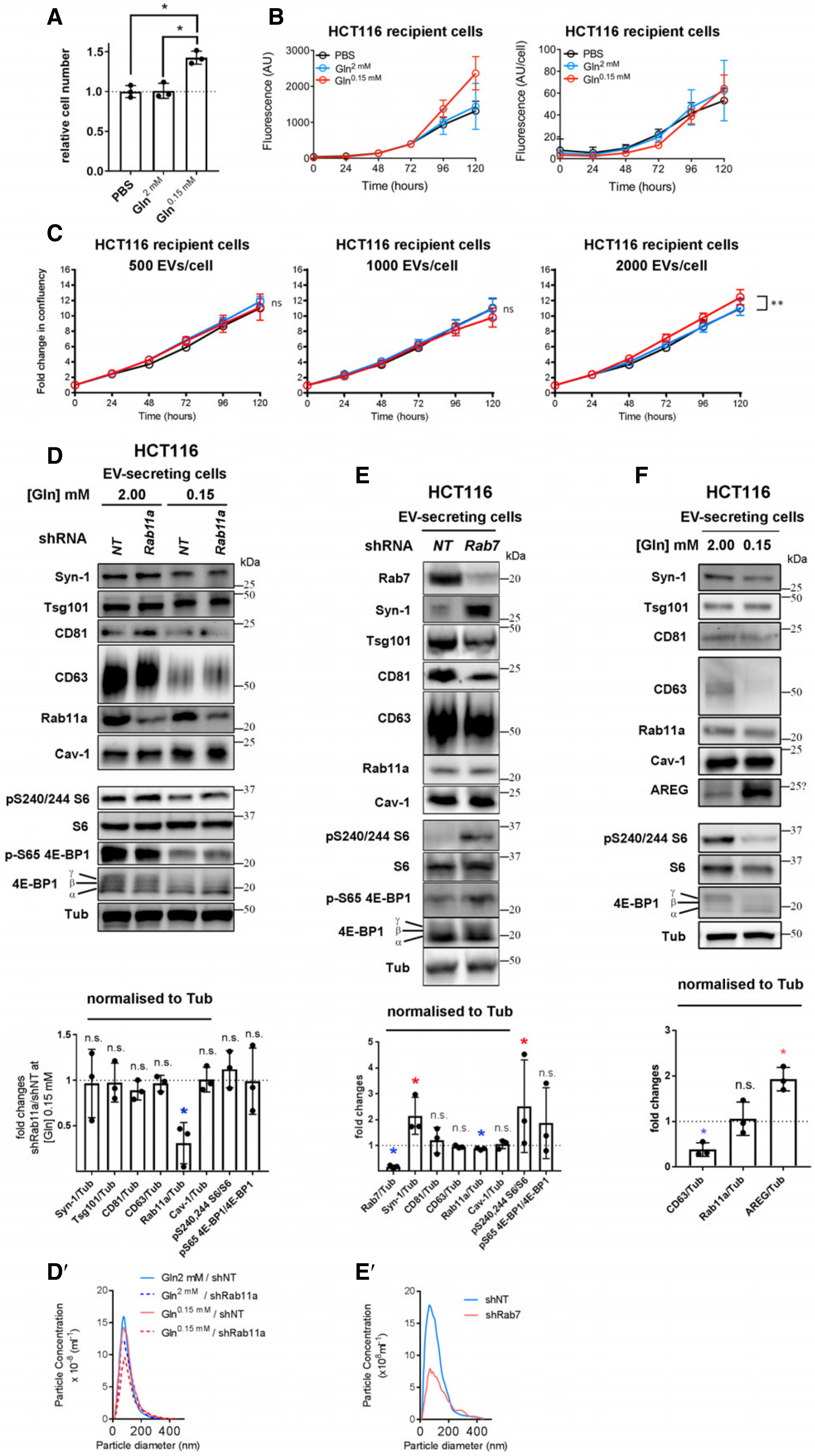
A
relative cell number
PBS
Gln 2 mM
Gln 0.15 mM
B
HCT116 recipient cells
Fluorescence (AU)
Fluorescence (AU/cell)
Time (hours)
PBS
Gln2 mM
Gln0.15 mM
C
HCT116 recipient cells
500 EVs/cell
1000 EVs/cell
2000 EVs/cell
Fold change in confluency
Time (hours)
ns
**
D
HCT116
EV-secreting cells
[Gln] mM 2.00 0.15
shRNA NT Rab11a NT Rab11a
kDa
Syn-1
Tsg101
CD81
CD63
Rab11a
Cav-1
pS240/244 S6
S6
p-S65 4E-BP1
4E-BP1
Tub
normalised to Tub
fold changes shRab11a/shNT at [Gln] 0.15 mM
n.s.
Syn-1/Tub
Tsg101/Tub
CD81/Tub
CD63/Tub
Rab11a/Tub
Cav-1/Tub
pS240,244 S6/S6
pS65 4E-BP1/4E-BP1
E
HCT116
EV-secreting cells
shRNA NT Rab7
Rab7
fold changes
Rab7/Tub
F
[Gln] mM 2.00 0.15
AREG
AREG/Tub
D′
Gln2 mM / shNT
Gln2 mM / shRab11a
Gln0.15 mM / shNT
Gln0.15 mM / shRab11a
Particle Concentration x 10-8 (ml-1)
Particle diameter (nm)
E′
shNT
shRab7
Particle Concentration (x108ml-1)

**Figure EV5.**
