# Supplementary material for: Glutamine deprivation alters the origin and function of cancer cell exosomes
Source: EMBO J. 2020 Jul 28;39(16):e103009. doi: 10.15252/embj.2019103009 (PMC7429491; doi:10.15252/embj.2019103009)
Supplement: Supplementary file 3 — Movie EV1 [file EMBJ-39-e103009-s003.zip › EMBOJ-2019-103009_Movie_EV1_legend.pdf]

Movie EV1. Intraluminal vesicles are present in non-acidic compartments of *Drosophila* secondary cells (related to Fig 1)

Movie of Z-stack generated from super-resolution 3D-SIM images of a living SC expressing CD63-GFP (yellow) and labelled with LysoTracker Red® (magenta). It focuses on a single non-acidic compartment, which has intraluminal vesicles (ILVs) of varying size arranged in at least four clusters. ILV size is quantified in Fig EV1A.

Genotype of fly is *w; P[w⁺, UAS-CD63-GFP] P[w⁺, tub-GAL80ts]/+; dsx-GAL4/+*.

Scale bar is 1 µm. The Z-stack includes 51 sections at 0.125 µm intervals.
