# Supplementary material for: Glutamine deprivation alters the origin and function of cancer cell exosomes
Source: EMBO J. 2020 Jul 28;39(16):e103009. doi: 10.15252/embj.2019103009 (PMC7429491; doi:10.15252/embj.2019103009)
Supplement: Supplementary file 4 — Movie EV2 [file EMBJ-39-e103009-s004.zip › EMBOJ-2019-103009_Movie_EV2_legend.pdf]

**Movie EV2. Rab11 is not trafficked to the apical plasma membrane of *Drosophila* secondary cells (related to Fig EV1H)**

Movie of Z-stack generated from wide-field fluorescence transverse images of a living SC of a male fly expressing a YFP-Rab11 gene trap (yellow). Fig EV1H shows one of these images. Acidic compartments are marked by LysoTracker Red® (magenta). Unlike CD63-GFP, YFP-Rab11 is not localised to the apical plasma membrane. Punctate intraluminal vesicles are marked by YFP-Rab11 inside large Rab11-compartments (more clearly seen in Fig EV1H). YFP-Rab11 is also observed in the cytosol.

Scale bar is 5 µm. The Z-stack includes 27 sections at 0.5 µm intervals.
