# Supplementary material for: Glutamine deprivation alters the origin and function of cancer cell exosomes
Source: EMBO J. 2020 Jul 28;39(16):e103009. doi: 10.15252/embj.2019103009 (PMC7429491; doi:10.15252/embj.2019103009)
Supplement: Supplementary file 5 — Movie EV3 [file EMBJ-39-e103009-s005.zip › EMBOJ-2019-103009_Movie_EV3_legend.pdf]

Movie EV3. Shrb-GFP accumulates in microdomains at the surface of large non-acidic compartments in *Drosophila* secondary cells (related to Fig EV2G)

Movie of Z-stack generated from wide-field fluorescence images of a living SC after a 4 h pulse of Shrb-GFP. Acidic compartments are marked by LysoTracker Red® (magenta). Note the accumulation of Shrb-GFP in microdomains at the surface of non-acidic and acidic compartments. Fig EV2G shows a single Z-plane from this movie.

Scale bar is 5 µm. The Z-stack includes 56 sections at 0.2 µm intervals.
